# Polymorphism of Genes Potentially Affecting Growth and Body Size Suggests Genetic Divergence in Wild and Domestic Reindeer (*Rangifer tarandus*) Populations

**DOI:** 10.3390/genes15121629

**Published:** 2024-12-20

**Authors:** Anna A. Krutikova, Natalia V. Dementieva, Yuri S. Shcherbakov, Vasiliy V. Goncharov, Darren K. Griffin, Michael N. Romanov

**Affiliations:** 1 Russian Research Institute of Farm Animal Genetics and Breeding—Branch of the L. K. Ernst Federal Research Centre for Animal Husbandry, Pushkin 196601, Russia; anntim2575@mail.ru (A.A.K.); yura.10.08.94.94@mail.ru (Y.S.S.); 2 Department of Genetic and Reproductive Biotechnologies, Saint Petersburg State University of Veterinary Medicine, Saint Petersburg 196084, Russia; 3 Research Institute of Agriculture and Ecology of the Arctic—Branch of the Federal Research Center “Krasnoyarsk Science Center”, Norilsk 663302, Russia; wgoncharow@mail.ru; 4 School of Natural Sciences, University of Kent, Canterbury CT2 7NJ, UK; d.k.griffin@kent.ac.uk; 5 Animal Genomics and Bioresource Research Unit (AGB Research Unit), Faculty of Science, Kasetsart University, Chatuchak, Bangkok 10900, Thailand; 6 L. K. Ernst Federal Research Center for Animal Husbandry, Dubrovitsy, Podolsk 142132, Russia

**Keywords:** reindeer *Rangifer tarandus*, growth, body size, polymorphism, genes, *GHR*, *GH*, *LCORL*, *BMP2*, genetic divergence

## Abstract

**Background/Objectives**: A combination of increased human presence in the Arctic zone alongside climate change has led to a decrease in the number of wild reindeer (*Rangifer tarandus*). Studying the genetic potential of this species will aid in conservation efforts, while simultaneously promoting improved meat productivity in domestic reindeer. Alongside reducing feed costs, increasing disease resistance, etc., acquiring genetic variation information is a crucial task for domestic reindeer husbandry. This study thus identified highly informative molecular genetic markers usable for assessing genetic diversity and breeding purposes in reindeer. **Methods**: We analyzed gene polymorphism that may potentially affect animal growth and development in populations of wild (Taimyr Peninsula) and domestic reindeer, including Nenets and Evenk breeds. We screened these populations for polymorphisms by sequencing the *GH*, *GHR*, *LCORL* and *BMP2* genes. **Results**: Following generation of gene sequences, we compared the alleles frequency in the surveyed populations and their genetic divergence. Some loci lacked polymorphism in wild reindeer, unlike domestic breeds. This could suggest a selection-driven microevolutionary divergence in domestic reindeer populations. An isolated domestic population from Kolguyev Island appeared to be genetically remote from continental reindeer. **Conclusions**: Molecular genetic markers associated with economically important traits in reindeer can be further developed using the data obtained. Monitoring wild reindeer populations and better utilizing the genetic potential of domestic animals will depend on a panel of these marker genes. By using this marker panel, the amount of time spent on selection efforts will be greatly reduced to enhance meat performance during reindeer breeding.

## 1. Introduction

The wild reindeer (*R. tarandus* L. 1758) population in Eurasia is estimated to be over one million, but is declining [1] due to various factors. These include increased human presence (e.g., due to tourism) in the Arctic zone alongside climate changes that lead to a habitat deterioration, particularly in the quality of pasture lands [2,3,4]. In addition, overhunting, disease spread [5], the expansion of mining operations and the usage of land for breeding domestic reindeer [6,7] all contribute to this decline. To the best of our knowledge, reindeer is the only species in which the exchange of genetic material between wild and domesticated forms is not restricted [8,9]. The largest population of wild reindeer in Russia is localized on the Taimyr Peninsula, which has not undergone long periods of sharp decline [10].

Russia is home to two-thirds of the world’s domestic reindeer population [11]. In 1985, four breeds of reindeer were approved and entered into the State Register. These are the Nenets [12,13], Chukchi, Even and Evenk breeds. The most numerous of these is the Nenets breed that number over one million individuals. In the western territory of its distribution, the Nenets breed has contact with the wild and Sámi reindeer, in the north and south the breed has virtually no contact with wild reindeer and other domestic breeds. In the east, its contacts have been recorded with the Taimyr wild reindeer population and the Evenk breed [12]. Phenotypic differences between both wild and domestic reindeer are mostly due to habitat conditions. Reindeer grazing in the tundra (e.g., the Nenets breed) are significantly smaller in size than those living in the forest-tundra, taiga, or mountainous regions (e.g., the Evenk breed) [14,15]. The size of an animal is of great importance for survival in the harsh Arctic conditions, where there are many predatory animals. In addition, hunters prefer larger individuals, which leads to a decrease in the survival of wild populations.

Research by Pokharel et al. [16] demonstrated that genes, which differentiate reindeer populations, are associated with evolutionary processes. This may indicate genetic diversity across all populations in genes associated with animal growth and size. Recently, studies on reindeer have been focused on the generation of a complete genome assembly, which will significantly advance our knowledge of the variability of populations belonging to the ruminant species most adapted to extreme habitat conditions [16,17,18,19]. As a result of whole genome sequencing, a large set of *R. tarandus* scaffolds and whole chromosome sequences is available in the NCBI databases [20]; this makes it possible to compare sequenced regions of individual genes (e.g., growth hormone receptor (*GHR*) gene [21,22]) with information contained within whole genome sequence databases.

In our previous studies [23,24,25], we preliminarily evaluated the variability of the *GH* (growth hormone), *LCORL* (ligand dependent nuclear receptor corepressor like) and *BMP2* (bone morphogenetic protein 2) gene regions that are, most likely, important for the formation of reindeer body size. The current investigation provides a further information on the partial sequencing and, for the first time, polymorphism of the *GHR* gene region, supplemented by a comparative analysis of polymorphic variants of the above studied genes, including a novel *LCORL* indel.

## 2. Materials and Methods

### 2.1. Sample Collection and Genomic DNA Extraction

**Figure 1:**
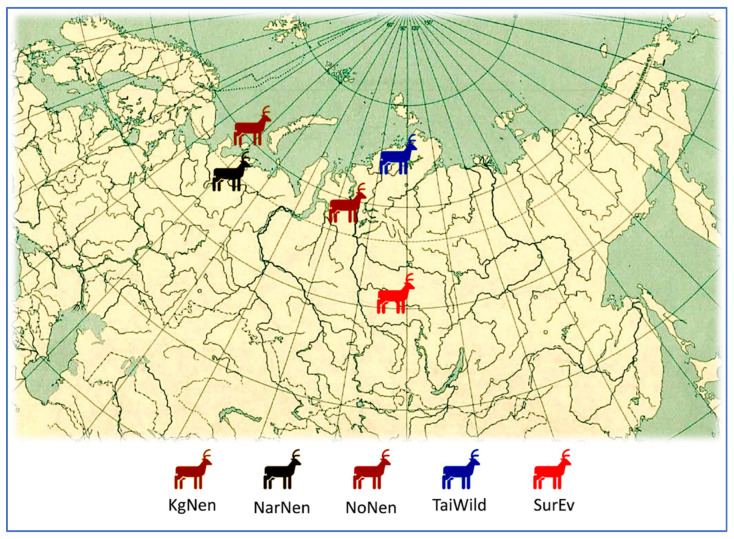
Sampling sites of the reindeer (*Rangifer tarandus*) in the Far North of Russia. Populations/breeds designation: KgNen, Nenets breed, Kolguyev Island; NarNen, Nenets breed, Naryan-Mar; NoNen, Nenets breed, Nosok; TaiWild, wild reindeer, Taimyr Peninsula; SurEv, Evenk breed, Surinda.

The experiments were conducted in the Laboratory of Molecular Genetics, Russian Research Institute of Farm Animal Genetics and Breeding (RRIFAGB). The material for the studies was DNA isolated from samples of biological tissues of wild and domestic reindeer collected at five sites in the Far North of Russia (Figure 1).

**Figure 2:**
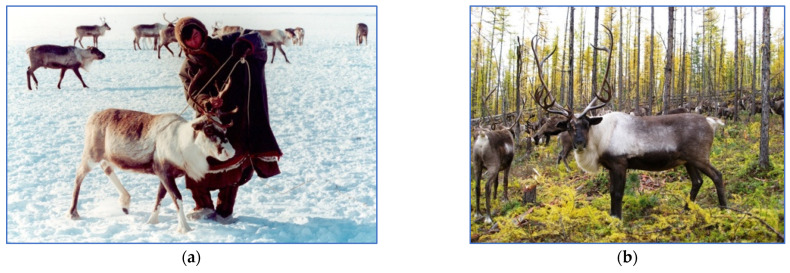

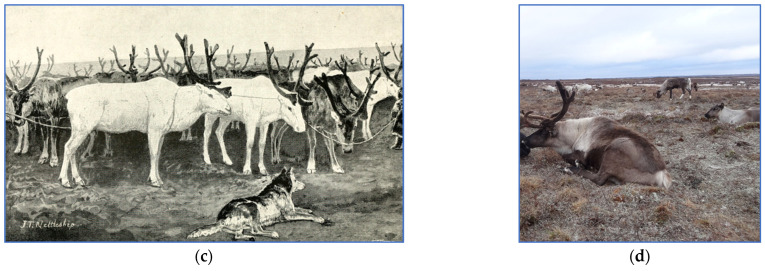
Reindeer (*Rangifer tarandus*) in the Far North of Russia: (**a**) Nenets breed that has a smaller body size, (**b**) a larger Evenk breed, (**c**) Nenets breed on Kolguyev Island and (**d**) reindeer in the Taimyr Peninsula. Credit: (**a**,**b**,**d**) own authors’ photographs; (**c**) https://commons.wikimedia.org/wiki/File:Ice-bound_on_Kolguev_-_a_chapter_in_the_exploration_of_Arctic_Europe_to_which_is_added_a_record_of_the_natural_history_of_the_island_(1895)_(14779560514).jpg (accessed on 27 November 2024) (by J.T. Nettleship, 1895; no known copyright).

Blood samples taken from the jugular vein; ear notches as well as parts of muscle tissue of shot wild reindeer were used to isolate DNA. DNA extraction was carried out using the conventional phenol method. For the *GHR* and *GH* [23] gene analyses, the following samples were obtained (Figure 1): from the tissues of wild reindeer from the Taimyr Peninsula (TaiWild; *n* = 10; Figure 2c), as well as domestic breeds, including the Nenets breed (Figure 2a) from the village of Nosok (NoNen; *n* = 10), vicinity of the Naryan-Mar city (NarNen; *n* = 11) and Kolguyev Island (KgNen; *n* = 11) and the Evenk breed (Figure 2b) from the village of Surinda (SurEv; *n* = 6). For the *LCORL* [24] and *BMP2* [25] gene analyses, a different set of samples was employed, including the wild population of the Taimyr Peninsula (*n* = 20), the Nenets breed (*n* = 20) and the Evenk breed (*n* = 20).

### 2.2. PCR, Gene Sequencing and Computational Analyses

Initial lab work was performed to optimize the PCR amplification conditions and select annealing temperatures for each primer pairs specific for certain regions of the *GHR*, *GH*, *LCORL* and *BMP2* genes used in the comparative analysis within the framework of this study (Table 1). The PCR primers were synthesized by Syntol LLC (Moscow, Russia).

Purification of PCR products for further sample preparation before sequencing was performed using the commercial ExoSAP-IT Express enzymatic purification kit for PCR products (Affymetrix, Santa Clara, CA, USA).

Sanger sequencing of the generated amplicons was performed using an Applied Biosystems 3500 Genetic Analyzer using the commercial BigDye^®^ Terminator v3.1 Sequencing Standard Kit (Applied Biosystems, Waltham, MA, USA) according to the manufacturer’s protocol. The obtained partial gene sequences were aligned and analyzed using Mega 6 software [26,27,28] and deposited in GenBank. Supplementary Information S1 contains the raw genotyping data.

Further analysis of gene sequences was carried out within the bioinformatic environment of the NCBI genetic databases [29]. Allele frequencies were calculated at each locus and in each population (Supplementary Information S1) and used for hierarchical clustering of the populations/breeds studied. The latter was performed using the Phantasus web application [30]. Accordingly, Euclidean distances were computed for columns of the allele frequency matrices (with the *average* option selected for the clustering method) and hierarchical clustering trees were subsequently generated. As the genetic diversity statistics at SNP loci in the *GHR* and *GH* genes, we computed observed heterozygosity (*H_O_*), expected heterozygosity (*H_E_*), unbiased expected heterozygosity (*_U_H_E_*) [31] and rarefied allelic richness (*A_R_*) [32] using the R library diveRsity package [33] in the five studied populations, i.e., TaiWild, SurEv, NoNen, NarNen and KgNen. Additionally, we ranked these populations in a simplified form by the diversity indicator values from 1 (the lowest one) to 5 (the highest one) and calculated the corresponding mean rank values. Similar genetic diversity computations and ranking were performed for SNP loci in the *LCORL* and *BMP2* genes in one wild population and two domestic breeds, Nenets and Evenk.

## 3. Results and Discussion

### 3.1. GHR and GH Gene Polymorphisms

In this study, we investigated the *GHR* gene polymorphism in wild and domestic reindeer; to the best of our knowledge, it is the first time that this has been achieved (Table 2, Supplementary Information S1). This is one of the key genes responsible for growth formation in animals [22]. The obtained data on the frequency of occurrence of three *GHR* gene polymorphic variants in wild and domestic reindeer differed slightly (Table 2), except the Evenk breed of domestic reindeer (SurEv) that distinguishes from other populations by its greater height and a much lower frequency of the single nucleotide polymorphism (SNP) GHR3 allele C (0.08). Another exception was the wild reindeer population (Tai) that had a greater frequency of the SNP GHR1 allele A (0.80). The data we obtained as a result of sequencing the *GHR* gene had a few discrepancies with the previously deposited sequence of exon 10 of this gene available for the *R. tarandus* [21,22]. Data on the detected genetic differences at the nucleotide alignment level are provided in Supplementary Information S2a. The importance of identifying *GHR* gene polymorphism is due to the fact that its functioning deficiency can cause smaller height phenotypes [34].

**Figure 3:**
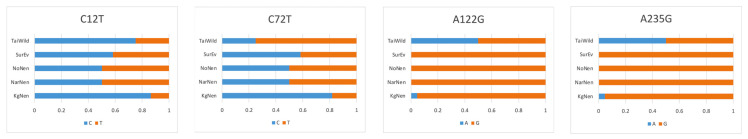
Occurrence frequencies of four SNP alleles found in the *GH* gene [23]. Populations/breeds designation: TaiWild, wild reindeer, Taimyr Peninsula; SurEv, Evenk breed, Surinda; NoNen, Nenets breed, Nosok; NarNen, Nenets breed, Naryan-Mar; KgNen, Nenets breed, Kolguyev Island.

In our previous studies [23], we also sequenced regions of exons 2 and 3 in the *GH* gene. Supplementary Information S2b contains information on the genetic changes identified at the nucleotide alignment level. This was carried out for a total of 48 reindeer from the same five populations spread in northern Russian Eurasia and showed the presence of four SNPs, i.e., C12T, C72T, A122G and A235G (Figure 3, Supplementary Information S1). It should be noted that in mammals, the structure of pituitary GH is generally strictly conserved [35,36]. However, in primates and artiodactyls, the rate of evolution in this gene has increased dramatically (25–50-fold), so that the *GH* sequences of humans and ruminants differ significantly from the sequences of other mammals [35,36]. At the same time, the *GH* gene sequence in red deer (*Cervus elaphus*) differs significantly from that of other ruminants [37]. Wild reindeer from the Taimyr Peninsula in our studies [23] had a higher level of polymorphism for all four SNPs (Figure 3). Unlike wild reindeer, domestic breeds had similar homozygotes with a low minor allele frequency for two of the four polymorphisms.

When analyzing the genetic diversity indicators for a total of seven SNP loci in *GHR* and *GH* genes (Table 3), we noted that the SurEv population tended to have the lowest *H_E_*, *_U_H_E_* and *A_R_* values, while the wild population (TaiWild) seemed to have the highest values. However, because of the overall mixed diversity pattern for the five populations and for the four indices, we also estimated the data in a simplified form using the population ranking (Table 3). Herewith, the SurEv population was least variable (M = 1.5) and the NarNen and TaiWild populations demonstrated the highest mean diversity rank (M = 4).

**Figure 4:**
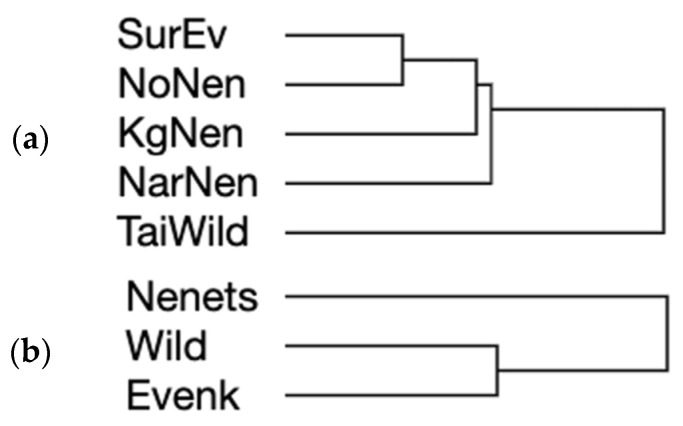
Hierarchical clustering dendrograms showing the genetic diversity differences between the studied reindeer populations/breeds. Hierarchical clustering trees were built using SNP genotyping data (**a**) at *GHR* (present study) and *GH* [23] loci, and (**b**) at *LCORL* (present study and [24]) and *BMP2* [25] loci. Populations/breeds designation: (**a**) SurEv, Evenk breed, Surinda; NoNen, Nenets breed, Nosok; KgNen, Nenets breed, Kolguyev Island; NarNen, Nenets breed, Naryan-Mar; TaiWild, wild reindeer, Taimyr Peninsula; (**b**) Nenets, Nenets breed; Wild, wild reindeer, Taimyr Peninsula; Evenk, Evenk breed.

A very similar genetic diversity difference pattern was observed when implementing the hierarchical clustering of the studied populations (Figure 4a) using the same set of diversity values (Table 3).

### 3.2. LCORL and BMP2 Gene Polymorphisms

**Figure 5:**
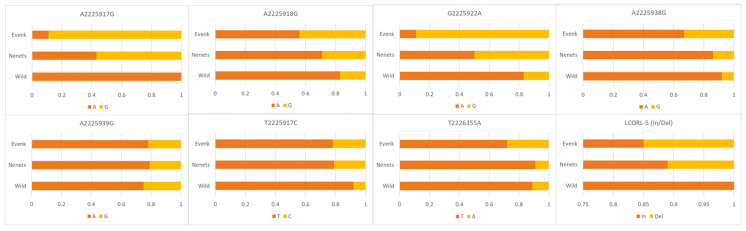
Occurrence frequencies of eight SNP alleles observed in the *LCORL* gene in domestics (of the Evenk and Nenets breeds) and the wild reindeer (present study and [24]).

One more important gene putatively influencing the formation of the skeletal size of animals is the *LCORL* gene [38,39,40]. In our studies [24], we sequenced exon 7 of the *LCORL* gene, where seven single-nucleotide substitution variants and one insertion/deletion polymorphism were identified (Figure 5, Supplementary Information S1; [24]). In particular, there was one previously undescribed 36-bp deletion detected, namely, in region 5 in exon 7 of the *LCORL* gene; this was found only in domestic reindeer.

The length of a novel deletion identified by us in exon 7 of the *LCORL* gene allows to detect its presence even by electrophoresis in an agarose gel. Analysis of the frequency of this deletion showed that such a mutation does not occur in the surveyed sampling of wild reindeer; accordingly, the frequency of the genotype for the alternative allele In/In in the wild population was 1.00. A lower frequency of the minor allele Del was observed in the analyzed sample of domestic reindeer of the Nenets breed and was 0.11. In reindeer of the Evenk breed, the frequency of the minor allele Del was slightly higher and was 0.15 (Figure 5).

**Figure 6:**
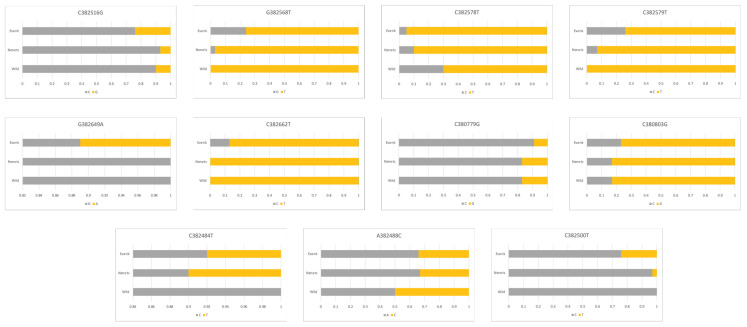
Occurrence frequency of 11 SNP alleles identified in the *BMP2* gene in domestics (of the Evenk and Nenets breeds) and the wild reindeer [25].

The *BMP2* gene is another gene associated with animal growth and antler formation in deer [41]. In our earlier studies [25], 11 polymorphic variants were found and studied in wild and domestic reindeer (Figure 6, Supplementary Information S1). However, few clear-cut differences were found between wild and domestic reindeer at first glance. It should be noted that there were slight differences in the allele frequencies in the Evenk breed, which is the largest in size among reindeer.

Using genotyping information for a total of 19 SNP loci in the *LCORL* and *BMP2* genes to analyze genetic diversity indices (Table 4), we established that the Nenets breed was inclined to show the lowest values of all the four statistics calculated. The Evenk breed and wild reindeer were prone to be more variable. This diversity pattern seemed to be similar using the simplified ranking form (Table 4) and hierarchical clustering (Figure 4b) procedures.

### 3.3. Divergence Estimation and Other General Considerations

**Figure 7:**
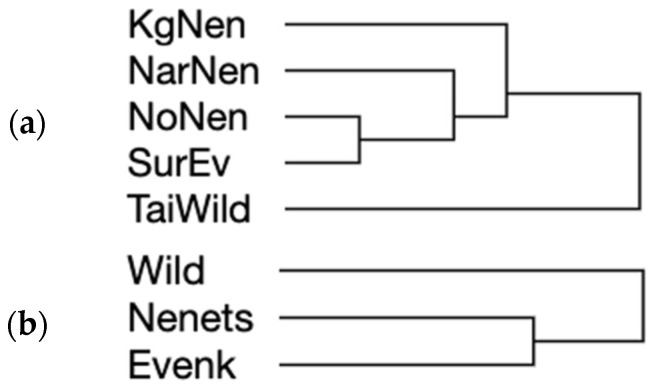
Genetic divergence plots based on occurrence frequencies of SNP alleles. Hierarchical clustering trees were built using SNP allele frequencies identified (**a**) at *GHR* (present study) and *GH* [23] loci, and (**b**) at *LCORL* (present study and [24]) and *BMP2* [25] loci. Populations/breeds designation: (**a**) KgNen, Nenets breed, Kolguyev Island; NarNen, Nenets breed, Naryan-Mar; NoNen, Nenets breed, Nosok; SurEv, Evenk breed, Surinda; TaiWild, wild reindeer, Taimyr Peninsula; (**b**) Wild, wild reindeer, Taimyr Peninsula; Nenets, Nenets breed; Evenk, Evenk breed.

Based on the allele frequencies at the analyzed SNP loci in the *GHR*, *GH*, *LCORL* and *BMP2* genes, we reconstructed the respective hierarchical clustering trees (Figure 7). Their topology suggests the genetic divergence between the wild and domestic reindeer populations/breeds. The wild reindeer was maximally distant from the domestics and the domestic populations/breeds formed one large cluster.

According to the occurrence frequencies of a total of seven SNP alleles in the *GHR* and *GH* genes (Figure 7a), the NoNen population of the Nenets breed was most closely related to the SurEv population of the Evenk breed, while the isolated KgNen population of the Nenets breed from Kolguyev Island was genetically most remote from the continental populations of two domestic breeds. In this context, the KgNen population was established in the 19th century (Figure 2c), originating from domestic reindeer brought to the island from the mainland about 200 years ago. In the 20th century, the number of domestic reindeer here reached around 20,000. Until 2012, the reindeer population remained at a relatively high level (~12,000), but then there was a mass mortality of animals. By 2015, 153 reindeer remained on the island [42,43]. Presently, the population is recovering (~2000 in 2021) [43]. Our findings were derived from the sampling before the mass mortality and do not reflect the current genetic diversity of the KgNen population (Table 3, Figure 4a). A systematic genetic monitoring of this population would provide crucial information for further preservation and exploitation of KgNen reindeer whose herding is the basis of local economy.

We also attempted to estimate genetic diversity and establish its varied pattern across the populations/breeds studied depending on the genes used for genotyping (Table 3 and Table 4, Figure 4). Wild reindeer was, most likely, characterized by a higher overall diversity, although, in contrast to domestic breeds, certain loci in wild reindeer lacked polymorphism. However, taking into account a rather limited number of sequenced animal samples per population, we would cautiously consider these diversity assessment patterns as preliminary, in that they require further investigation using larger population sampling sizes and more SNP markers.

The process of domestication of wild animals, including reindeer, inevitably leads to significant changes in their genome [44,45,46,47]. The longer the exposure of animal species, the more significant changes are fixed in their genes. The most significant changes occur in the genes of interest, i.e., genes involved in metabolic pathways in the process of formation of productive traits [18,48,49] that lead to increased growth, more meat, higher yield and quality of wool, increased milk production, etc. [50,51]. Mutations that spontaneously arise in both wild and domestic animals and lead to hypertrophy of quantitative traits have different ways of implementation in wild and domestic populations of the same species [52,53,54,55,56]. In domestic animals, such a mutation will be fixed due to artificial selection carried out by humans and further breeding work [57,58,59,60]. In a wild population, such mutations are more susceptible to elimination due to natural selection. The realization of the genetic potential of a mutation leading to hypertrophy of quantitative traits was associated, first of all, with increased feed consumption, and secondly, with its balance [61], which is not always feasible in natural conditions, as in the case of wild reindeer.

The current interest in the genetic study of reindeer is due to the fact that it is one of the few currently existing animals that are well-represented both in the wild and domesticated state. In addition, the reindeer is one of the most recently domesticated animals [62,63,64,65]. The main and practically the only type of productivity in reindeer is meat performance [66,67]. In Russia, when breeding reindeer, meat productivity is the main indicator; strength of constitution, growth, endurance to climatic conditions and draft work, and resistance to diseases are secondary [7]. In this regard, further study of the genetic characteristics of reindeer is required to understand the hereditary factors underlying the specific phenotypic and adaptive characteristics of this animal common in cold climates.

## 4. Conclusions

When introducing molecular genetic methods to study the characteristics of wild species populations and to the selection process in agricultural species, genes of interest responsible for adaptability, as well as dairy, meat and other productivity traits need to be thoroughly investigated [68,69]. From a whole range of genes playing a significant role in growth and muscle mass formation, potential candidates are *GHR*, *GH*, *LCORL* and *BMP2*. Here, we completed a preliminary comparative study of polymorphic regions of these genes in wild and domestic reindeer of Russia. Several SNPs and indels were identified, as was their occurrence in the genome of wild and domestic reindeer from different regions. The analysis of genes potentially associated with animal growth revealed features of the frequencies of polymorphic sequence variants that distinguish wild reindeer of the Taimyr Peninsula [70] from domestic reindeer breeds. Wild reindeer had no or fewer polymorphisms for a number of polymorphic variants, which may indicate microevolutionarily formed and selection-driven differences underlying the genetic divergence between wild and domestic reindeer populations. Domestic reindeer of the Far North of Russia had different levels of polymorphism for all identified SNPs, which is apparently the result of the selection process in populations. Herewith, the isolated population of Kolguyev Island is likely to be genetically more distant from continental domestic populations.

The obtained data can serve as a starting point for further developing molecular genetic markers associated with economically important traits in reindeer. A panel of such marker genes will be critical in monitoring wild reindeer populations and using the genetic potential of domestic animals more effectively. Application of this marker panel will significantly reduce the time spent on the selection process to improve meat performance in the course of reindeer breeding. The results reported here will be developed further to clarify the localization of polymorphisms in the reindeer genome and their use for the purpose of effective restoration of wild reindeer populations. Subsequent selection for the purposes of domestic breeding can then ensue.

## Figures and Tables

**Table 1:** PCR primers for analyzing the *GHR*, *GH*, *LCORL* and *BMP2* gene fragments.

Gene/Locus	Primers (F, Forward; R, Reverse)	Fragment Size, bp
*GHR*	Exon 10 F: TTTGTTAAATCAATTGTTGTGAG R: GTCGCATTGAGTACAAGGC	
		844
*GH*	Exons 2 and 3 F: GGAGAAGCAGAAGGCAACC R: CTCTGCCTGCCCTGGACT	
		382
*LCORL*	Exon 7 regions	
LCORL-5	F: CATCCAAGAAATTGATAGAA R: TTTCACAACCTGGGGACCTA	682, 646
LCORL-9	F: TTTTGAGTAAGACTGAGGGA R: GTGGTCTTCCATGGTGGTCT	657
LCORL-10	F: TCTTAGCAAACTGAACAAAA R: GCCAAGAAATTAGATTGTCCA	640
*BMP2*	Regions of exons 1 and 2	
BMP2-1 (exon 1)	F: TCGCGGATTACTAGGGACTCA R: GCGCAAGTTATTCTCCCTGC	705
BMP2-1 (exon 2)	F: GCGCTGTGTGTTTGGGTTAG R: AAAGCCAGGTTCGGAAAGGT	872

**Table 2:** Occurrence frequencies of SNP alleles identified in the *GHR* gene (present study).

SNPs and Alleles	Populations ^1^				
	KgNen	NarNen	NoNen	SurEv	TaiWild
GHR1					
A	0.55	0.59	0.65	0.58	0.80
G	0.45	0.41	0.35	0.42	0.20
GHR2					
C	0.64	0.55	0.85	0.83	0.70
T	0.36	0.45	0.15	0.17	0.30
GHR3					
C	0.41	0.41	0.20	0.08	0.30
T	0.59	0.59	0.80	0.92	0.70

^1^ Populations/breeds designation: TaiWild, wild reindeer, Taimyr Peninsula; SurEv, Evenk breed, Surinda; NoNen, Nenets breed, Nosok; NarNen, Nenets breed, Naryan-Mar; KgNen, Nenets breed, Kolguyev Island.

**Table 3:** Summary of genetic diversity statistics ^1^ (M ± SE) calculated in the studied reindeer populations based on SNP genotypes identified in the *GHR* (present study) and *GH* [23] genes.

Populations	*H_O_*	*H_E_*	*_U_* *H_E_*	*A_R_*	
KgNen	0.26 ± 0.05	0.31 ± 0.07	0.32 ± 0.07	1.77 ± 0.09	
NarNen	0.44 ± 0.16	0.35 ± 0.09	0.37 ± 0.09	1.71 ± 0.18	
NoNen	0.37 ± 0.17	0.29 ± 0.08	0.30 ± 0.09	1.65 ± 0.17	
SurEv	0.28 ± 0.14	0.27 ± 0.08	0.29 ± 0.09	1.60 ± 0.17	
TaiWild	0.23 ± 0.02	0.41 ± 0.02	0.44 ± 0.03	1.95 ± 0.01	
	Ranks				M
SurEv	3	1	1	1	1.5
NoNen	4	2	2	2	2.5
KgNen	2	3	3	4	3
NarNen	5	4	4	3	4
TaiWild	1	5	5	5	4

^1^ M, mean value; SE, standard error; *H_O_*, observed heterozygosity; *H_E_*, expected heterozygosity; *_U_H_E_*, unbiased expected heterozygosity; *A_R_*, rarefied allelic richness.

**Table 4:** Summary of genetic diversity statistics ^1^ (M ± SE) computed for the studied wild reindeer and domestic breeds based on SNP genotypes identified in the *LCORL* (present study and [24]) and *BMP2* [25] genes.

Populations	*H_O_*	*H_E_*	*_U_* *H_E_*	*A_R_*	
Wild	0.32 ± 0.09	0.19 ± 0.05	0.19 ± 0.05	1.47 ± 0.12	
Nenets	0.16 ± 0.08	0.11 ± 0.05	0.11 ± 0.05	1.29 ± 0.11	
Evenk	0.28 ± 0.08	0.24 ± 0.05	0.24 ± 0.05	1.64 ± 0.12	
	Ranks				M
Wild	3	2	2	2	2.25
Nenets	1	1	1	1	1
Evenk	2	3	3	3	2.75

^1^ M, mean value; SE, standard error; *H_O_*, observed heterozygosity; *H_E_*, expected heterozygosity; *_U_H_E_*, unbiased expected heterozygosity; *A_R_*, rarefied allelic richness.

## Data Availability

The data contributions presented in this study are included in the article and Supplementary Materials. The raw sequencing data presented in the study is openly available in GenBank at: https://www.ncbi.nlm.nih.gov/genbank/ (accessed on 27 November 2024).

## Supplementary Materials

The following supporting information can be downloaded at: https://www.mdpi.com/article/10.3390/genes15121629/s1, Supplementary Information S1: Raw genotyping data, calculation of allele frequencies, and construction of frequency diagrams; Supplementary Information S2: Sequence alignments for the *GHR* (a) and *GH* (b) genes.
