# Supplementary material for: Polymorphism of Genes Potentially Affecting Growth and Body Size Suggests Genetic Divergence in Wild and Domestic Reindeer (Rangifer tarandus) Populations
Source: Genes (Basel). 2024 Dec 20;15(12):1629. doi: 10.3390/genes15121629 (PMC11675441; doi:10.3390/genes15121629)
Supplement: Supplementary file 1 [file genes-15-01629-s001.zip › Supplementary Information S2.pdf]

Supplementary Information S2: Sequence alignments for the *GHR* (a) and *GH* (b) genes

(**a**) Query: None Query ID: lcl|Query_1089601 Length: 844
>Rangifer tarandus growth hormone receptor (GHR) gene, exon 10 and partial cds
Sequence ID: DQ062724.1 Length: 900
Range 1: 1 to 828
Score:1471 bits(796), Expect:0.0,
Identities:817/828(99%), Gaps:3/828(0%), Strand: Plus/Plus

```
Query  20   GARGGAAAATTAGAAGAGGTGAATACAATCTTAGCCATTCAAGACAACTATAAACACGAA  79
            || |||||||||||||||||||||||||||||||||||||| ||||||||||||||||||
Sbjct  1    GAAGGAAAATTAGAAGAGGTGAATACAATCTTAGCCATTCATGACAACTATAAACACGAA  60

Query  80   TTCTACAATGATGACTCTTGGGTTGAATTTATTGAACTAGATATTGATGAC---GATGAA  136
            |||||||||||||||||||||||||||||||||||||||||||||||||||   ||||||
Sbjct  61   TTCTACAATGATGACTCTTGGGTTGAATTTATTGAACTAGATATTGATGACCCTGATGAA  120

Query  137  AAGACTGAAGGTTCAGACACAGACAGACTTCTGAGCAATGACCCTGAAAAATCACTCAAT  196
            ||||||||||| ||||||||||||||||||||||||||||||||||||||||||||||||
Sbjct  121  AAGACTGAAGGGTCAGACACAGACAGACTTCTGAGCAATGACCCTGAAAAATCACTCAAT  180

Query  197  ATCTTTGGGGCAAAAAATGACGACTYTGGGCGTACCAGCTGTTACGAACCTGACATTCTA  256
            ||||||||||||||  ||||||||| ||||||||||||||||||||||||||||||||||
Sbjct  181  ATCTTTGGGGCAAAGGATGACGACTCTGGGCGTACCAGCTGTTACGAACCTGACATTCTA  240

Query  257  GAGACTGATTTCCATGTCAGYGACATGTGCGATGGTACCTCAGAGGTTGCTCAGCCACAA  316
            |||||||||||||||||||| |||||||||||||||||||||||||||||||||||||||
Sbjct  241  GAGACTGATTTCCATGTCAGTGACATGTGCGATGGTACCTCAGAGGTTGCTCAGCCACAA  300

Query  317  AGGTTAAAAGGGGAAGCAGATATCTTGTGCCTTGATCAGAAGAATCAAAATAACTCACCT  376
            ||||||||||||||||||||||||||||||||||||||||||||||||||||||||||||
Sbjct  301  AGGTTAAAAGGGGAAGCAGATATCTTGTGCCTTGATCAGAAGAATCAAAATAACTCACCT  360

Query  377  TCTAATGATGCTGCCCCTGCTAGCCAGCAGCCCAGTGTTATCCTAGTAGAGGAAAACAAA  436
            ||||||||||||||||||||||||||||||||||||||||||||||||||||||||||||
Sbjct  361  TCTAATGATGCTGCCCCTGCTAGCCAGCAGCCCAGTGTTATCCTAGTAGAGGAAAACAAA  420

Query  437  CCAAGACCACTTCTTATTGGTGGAACTGAGTCAACTCATCAAGCTGTCCATACACAGCTC  496
            ||||||||||||||||||||||||||||||||||||||||||||||||||||||||||||
Sbjct  421  CCAAGACCACTTCTTATTGGTGGAACTGAGTCAACTCATCAAGCTGTCCATACACAGCTC  480

Query  497  AGCAATCCAAGTTCATTGGCAAACATTGATTTTTATGCCCAGGTAAGCGACATTACACCA  556
            | ||||||||||||||||||||||||||||||||||||||||||||||||||||||||||
Sbjct  481  AACAATCCAAGTTCATTGGCAAACATTGATTTTTATGCCCAGGTAAGCGACATTACACCA  540

Query  557  GCAGGGAATGTGGTCCTTTCCCCAGGCCAAAAGAATAAGACTGGGAACCCCCAGTGTGAC  616
            ||||||||||||||||||||||||||||||||||||||||||||||||||||||||||||
Sbjct  541  GCAGGGAATGTGGTCCTTTCCCCAGGCCAAAAGAATAAGACTGGGAACCCCCAGTGTGAC  600

Query  617  ATGCACCCAGAAGTGGTCACACCCTGCCAAGCAAACTTCATCATGGACAACGCTTACTTC  676
            ||||||||||||||||||||||||||||||||||||||||||||||||||||||||||||
Sbjct  601  ATGCACCCAGAAGTGGTCACACCCTGCCAAGCAAACTTCATCATGGACAACGCTTACTTC  660

Query  677  TGCGAGGTAGACGCCAAAAAGTACATTGCCCTGGCCCCTCACGTCGAGGCTGAATCACAT  736
            ||||||||||||||||||||||||||||||||||||||||||||||||||||||||||||
Sbjct  661  TGCGAGGTAGACGCCAAAAAGTACATTGCCCTGGCCCCTCACGTCGAGGCTGAATCACAT  720

Query  737  GTAGAGCCAAGCTTTAACCAGGAAGACATTTACATCACCACAGAAAGCCTTACCACTACA  796
            ||||||||||||||||||||||||||||||||||||||||||||||||||||||||||||
Sbjct  721  GTAGAGCCAAGCTTTAACCAGGAAGACATTTACATCACCACAGAAAGCCTTACCACTACA  780

Query  797  GCTGGGAGGTCGGGGACAGCAGAACATGTTCCAAGTTCTGAGATACCT  844
```

```
                ||||||||||||||||||||||||||||||||||||||||||||||||
Sbjct  781  GCTGGGAGGTCGGGGACAGCAGAACATGTTCCAAGTTCTGAGATACCT  828
```

(**b**) Query: None Query ID: lcl|Query_3011223 Length: 382
>Rangifer tarandus isolate US061 RanTarSib_scaffold_5875, whole genome shotgun sequence
Sequence ID: PVIN010002941.1 Length: 167893
Range 1: 83259 to 83640
Score:691 bits(374), Expect:0.0,
Identities:378/382(99%), Gaps:0/382(0%), Strand: Plus/Minus

```
Query  1      CAGGTGGTGGGYGCCTTCCCGGCCATGTCCTTGTCCGGCCTGTTTGCCAACGCTGTGCTC  60
              ||||||||||| ||||||||||||||||||||||||||||||||||||||||||||||||
Sbjct  83640  CAGGTGGTGGGCGCCTTCCCGGCCATGTCCTTGTCCGGCCTGTTTGCCAACGCTGTGCTC
83581

Query  61     CGGGCTCAGCAYCTGCATCAGCTGGCTGCTGACACCTTCAAAGAGTTTGTAAGCTCCCCA  120
              ||||||||||| ||||||||||||||||||||||||||||||||||||||||||||||||
Sbjct  83580  CGGGCTCAGCACCTGCATCAGCTGGCTGCTGACACCTTCAAAGAGTTTGTAAGCTCCCCA
83521

Query  121    GRGATGCATCCTAGGGGTGGGGAGGCAGGAAGGGGTGAATCTGCGCCCCCTCCACAGAGT  180
              | ||||||||||||||||||||||||||||||||||||||||||||||||||||||||||
Sbjct  83520  GGGATGCATCCTAGGGGTGGGGAGGCAGGAAGGGGTGAATCTGCGCCCCCTCCACAGAGT
83461

Query  181    GAGGGGACACTGAGTTCAGCGGAATTTTATCCAAGTGAGGATGCGGTCAGGGGARCAGAA  240
              |||||||||||||||||||||||||||||||||||||||||||||||||||||| |||||
Sbjct  83460  GAGGGGACACTGAGTTCAGCGGAATTTTATCCAAGTGAGGATGCGGTCAGGGGAGCAGAA
83401

Query  241    ACGGGGGTGTGTGGGGTGGGGAGGGTTCCGAATAAGGCAGTGAGGGGAACCGCGCACCAA  300
              ||||||||||||||||||||||||||||||||||||||||||||||||||||||||||||
Sbjct  83400  ACGGGGGTGTGTGGGGTGGGGAGGGTTCCGAATAAGGCAGTGAGGGGAACCGCGCACCAA
83341

Query  301    CTTAGACCTGAGTGGGCCTGTTCTTCCCCCAGGAGCGCACCTACATCCCGGAGGGACAGA  360
              ||||||||||||||||||||||||||||||||||||||||||||||||||||||||||||
Sbjct  83340  CTTAGACCTGAGTGGGCCTGTTCTTCCCCCAGGAGCGCACCTACATCCCGGAGGGACAGA
83281

Query  361    GATACTCCATCCAGAACACCCA  382
              ||||||||||||||||||||||
Sbjct  83280  GATACTCCATCCAGAACACCCA  83259
```
